# Bisphenol A Suppresses Release of Adipose Hormone: Exposure May Contribute to Metabolic Syndrome

**Published:** 2008-12

**Authors:** Tanya Tillett

Bisphenol A (BPA), a chemical used in the manufacture of numerous consumer products, is ubiquitous throughout the environment, and its widespread presence in human serum has been well documented. Although animal research indicates that BPA can alter several metabolic functions, interpretation of human data has been more controversial. A new study now presents evidence confirming that exposure of human adipose tissue and isolated fat cells to environmentally relevant levels of BPA suppresses release of the hormone adiponectin **[*EHP*** **116:1642–1647; Hugo et al.]**.

A high-calorie diet and sedentary lifestyle have both traditionally been linked to metabolic syndrome—the presence of a constellation of metabolic risk factors including insulin resistance, hypertension, and elevated blood sugar and lipid levels—but researchers are now examining environmental factors as additional causes. Adiponectin increases insulin sensitivity and reduces tissue inflammation, so suppression of its release could lead to insulin resistance and increased susceptibility to metabolic syndrome, the authors write.

The study examined three types of adipose tissue samples taken during breast reduction, abdominoplasty, and gastric bypass surgery. The research team incubated each type of tissue for 6 hours in BPA or estradiol (E_2_), an endogenous human estrogen. They used enzyme-linked immunosorbent assay to measure secreted adiponectin. They also used quantitative real-time polymerase chain reaction to compare the expression of estrogen receptors and estrogen-related receptors in these tissues.

In all three tissue types, exposure to low-nanomolar concentrations of BPA suppressed adiponectin as effectively or more effectively compared with equimolar concentrations of E_2_. The authors also showed that the dose response to BPA was nonmonotonic, meaning lower doses caused different effects than higher doses. Finally, they report for the first time similar mRNA expression levels for several estrogen receptors in visceral adipose tissue, although the role of these receptors in the suppressive nature of BPA and E_2_ has yet to be determined.

The results of the data are limited by the relatively small sample size in each tissue category, as well as the potential unknown effects of age or obesity on tissue responsiveness. However, the authors write that their data present clear evidence that BPA suppresses adiponectin, potentially leading to a much higher risk of developing metabolic syndrome and its resultant adverse health effects. They conclude that with BPA’s persistence in the environment, more research should be done to determine the mechanism by which the chemical suppresses adiponectin.

